# Heavy Metal Contamination and Ecological Risk Assessment in Soils and Sediments of an Industrial Area in Southwestern Nigeria

**DOI:** 10.5696/2156-9614-8.19.180906

**Published:** 2018-08-21

**Authors:** Tesleem O. Kolawole, Akinade S. Olatunji, Mustapha T. Jimoh, Olugbenga T. Fajemila

**Affiliations:** 1 Department of Geological Sciences, Osun State University, Osogbo, Nigeria; 2 Department of Geology, University of Ibadan, Ibadan, Nigeria; 3 Department of Earth Sciences, Ladoke Akintola University of Technology Ogbomosho, Nigeria

**Keywords:** ecological risk index, modified pollution index, contamination degree, industrial area

## Abstract

**Background.:**

Increased growth of industrial activities, especially in urban centers, is one of the main sources of toxic substances in Nigeria. The level of these impacts is not well known. Soil and sediment samples from one such industrial area were examined for their mineralogical composition and heavy metals contents in order to assess the level of contamination and potential ecological risk status.

**Methods.:**

Mineralogical composition of the media and their heavy metals concentrations were determined using X-ray diffractometry and inductively coupled plasma-mass spectrometry methods, respectively. Ecological risk assessment was carried out using single (contamination factor, geo-accumulation index, enrichment factor) and multi-elemental (contamination degree, pollution index and modified pollution index) standard indices.

**Results.:**

The average heavy metal concentrations in soils and sediments followed the order magnesium (Mn) > chromium (Cr) > lead (Pb) > copper (Cu) > cadmium (Cd) > cobalt (Co) > nickel (Ni), with corresponding values for soils and sediments of 324.3, 79.9, 66.1, 40.7, 14.3, 9.1, 6.8 mg kg^−1^ and 266.8, 78.6, 40.6, 39.8, 12.9, 8.4, 4.6 mg kg^−1^, respectively. Principal component (PC) analysis of the results indicated three main sources of metals (industrial, vehicular activities and geogenic input). Evaluated contamination factor (Cf), enrichment factor (E*f*) and geo-accumulation index (Igeo) revealed very high contamination for Pb, Cd and Cu in all of the samples, with calculated pollution index (PI) and modified pollution index (MPI) revealing that all the samples were severely polluted. Calculated potential ecological risk factor (ER^i^) within the industrial area demonstrated a strong potential ecological risk for Cd, Pb and Cu.

**Conclusions.:**

Activities in the industrial area have affected the quality of the analyzed environmental media, with possible detrimental health consequences. Regular environmental monitoring of the industrial area and the formulation of appropriate policies that support reduction of contamination are strongly recommended. However, due to the limitations of comparing site samples with a single control sample in this work, further study is recommended to compliment this preliminary study.

**Competing Interests.:**

The authors declare no competing financial interests

## Introduction

Heavy metals accumulation in soils, sediments and their subsequent release to ground or surface water poses an environmental threat. The extent of heavy metals contamination in these media is dependent on their sources, redox conditions, microbial activities and the physicochemical properties of solid and aqueous phases.^1^ The growth of heavy industries globally is a major source of high concentrations of heavy metals such as lead, zinc, copper, vanadium, chromium and molybdenum. The different sources of industrial inputs include industrial wastewater discharges, sewage wastewater, fossil fuel combustion, land deposition from landfills, atmospheric deposition and agrochemical inputs.^2–7^

Topsoil and stream sediment in the vicinity of industrial activities have often been found to be significantly contaminated with heavy metals. These metals, especially lead (Pb), pose a significant health hazard, particularly to children, who are the most susceptible to lead toxicity.^8^ In addition, weathering of rocks and the associated release of major and trace elements due to chemical changes and mineral alterations have been reported as sources of geogenic soil contamination.^9–11^

Different industrial pollutants are discharged into workplace and neighborhood environments in the form of particulate matter, which in turn settle on soil, and through direct discharge of industrial waste product directly into the immediate environment and nearby water bodies as industrial effluents.^12–16^ These practices and many more are common in cities of developing nations such as Ibadan, Southwestern Nigeria.

In Ibadan, industries are located close to residential areas and workers and residents are potentially exposed to released pollutants. Most industries in Ibadan lack effluent treatment plants and they discharge their effluents directly into water bodies without adequate treatment.^16^ Effluents are also directly discharged into surrounding rivers (Ona and Alaro) and these effluents are laden with chemical contaminants. These rivers also serve as alternative water sources for domestic, drinking and irrigation purposes.^20^,^21^ Local industries include soft drink bottling companies, confectioneries and diapers factories, and their products and raw materials have been reported to contain some heavy metals at values higher than tolerable levels.^17–19^ As river sediments serve as a sink for heavy metals, it is important to have reliable information on the quality of river sediment for effective planning and management.

Various methods have been developed for the assessment of heavy metals risk. The most important is the potential ecological risk index, as it is the only method that considers both concentrations and toxic response factors of heavy metals.^22^ To address this issue, Hakanson developed the potential ecological risk index, which introduces a toxic response factor for a given substance and thus can be used to evaluate the combined pollution risk to an ecological system.^23–26^

The present study aimed to determine the concentration and distribution of some heavy metals in soils and sediments in the study area, identify the sources of heavy metals using multivariate analyses, and evaluate the potential ecological risk levels of some heavy metals by applying the potential risk index method.

**Abbreviations**
*C*_*d*_: Contamination degree
*Er*^*i*^: Ecological risk factor
*Igeo*: Geo-accumulation index
*MPI*: Modified pollution index
*PC*: Principal component
*PI*: Pollution index

## Methods

**Figure 1:**
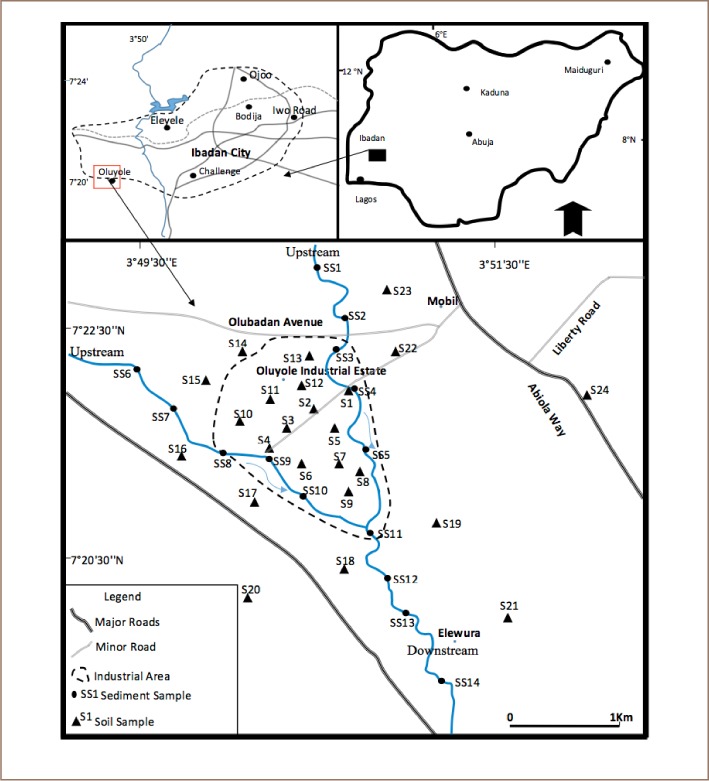
Map of the study area showing sample locations

Ibadan city is one of the largest cities in Nigeria, with a total area of 7434 km^2^ and a population over 3.5 million.^27^ It is also a major industrial and economic center. The prominent industries are situated within the Oluyole Industrial Estate, which is located in the southwestern part of the city. This area accommodates several large and medium-scale industries. These industries have been in operation for over three decades and are major pollution point sources. In addition to the waste they generate, these industries utilize heavy-duty machines, powered by heavy duty generators, which are also major sources of atmospheric pollutants. Other activities evident in the area include traffic from haulage vehicles and indiscriminate wood burning. The Ona and Alaro rivers drain the area, flowing southerly and draining the premises of most of factories (*Figure 1*) where they receive direct effluent discharges from the factories.

**Figure 2:**
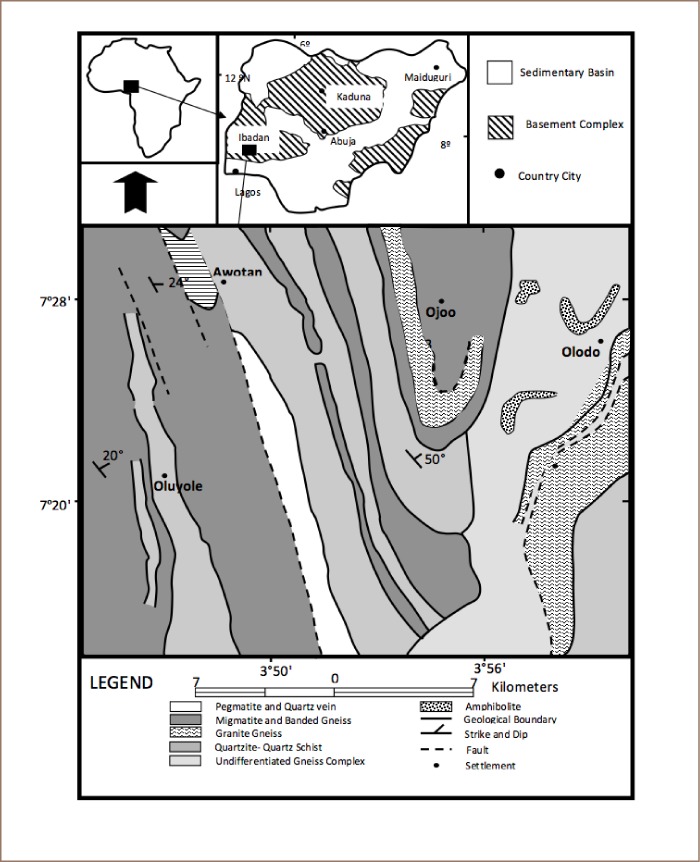
Geological map of the study area (after Okunlola et al., 2009).^28^

Geologically, the study area is underlain by rocks of both igneous and metamorphic origins. The dominant rock types are quartzites of the metasedimentary series and the migmatite complex made up of banded gneisses, augen gneisses and migmatites (*Figure 2*).^12^,^28^,^29^ These rocks are intruded by pegmatite, quartz veins, aplite and dioritic dykes. Minor rocks of substantial coverage include the amphibolites. In many places the rocks are overlain by very thick weathered regolith with few outcrops.

### Sample collection

Twenty-four (24) composite soil samples were collected at depths ranging from 0–15 cm within (13 samples) and outside (11 samples) the industrial area. The samples were collected based on accessibility. This constraint limited the sampling to opportunity samples, resulting in uncertainty as to the representativeness of the samples over the industrial area. One control sample was taken from an area devoid of industrial activities, but underlain by similar geological units. Soil samples were collected with a stainless-steel hand auger, stored in polyethylene bags and air-dried at 40°C for 48 hours.

Fourteen (14) composite stream sediments samples were collected along the Alaro River (the main river that passes through Oluyole Industrial Estate) and the rivulets forming its tributaries from December 21, 2015 to January 15, 2016. River sample locations were located at points before the river entered the industrial area and points after the river had drained the industrial area. These sampled points had a total length of 5 km (*Figure 1*). The sediments samples were then air dried.

### Sample preparation and analysis

All the dried soil and sediment samples were disaggregated in a porcelain mortar, sieved through a <75 μm polyethylene sieve to remove stones, plants roots, coarse materials, and other debris. Then 5 g of each of these samples were digested using aqua regia, and the processes were carried out adding 5 ml of nitric acid (Merck Suprapur 65%), 2 ml of hydrochloric acid (Merck Suprapur 36%) and 10 ml of ultra-pure water (18 MΩ cm^−1^ of specific resistivity) in a Pyrex tube and heated for 2 hour at 95°C on a hot plate. The extracted solution was filtered with a Whatman n°41 (WH1441-110) filter, completed to 50 ml with ultra-pure water and kept in pre-cleaned polyethylene bottles in the refrigerator until analysis. Elemental content of the digested samples was carried out with inductively coupled plasma mass spectrometry.

Quality assurance and quality control procedures were conducted by using standard reference materials: United States Geological Survey's Geochemical Exploration References Samples (GXR)-1, GXR-2, GXR-4 and GXR-6. Recoveries of the 8 observed heavy metals were between 93%–101% for nickel (Ni), 89%–103 for cadmium (Cd), 99%–103 for cobalt (Co), 93%–101% for copper (Cu), 88%–102% for iron (Fe), 94%–105% for Pb, 96%–103% for Magnesium (Mn), and 94%–106% for chromium (Cr). Duplicated samples were performed simultaneously for 20% of the soil samples, the standard deviation ranged within 5%, and blank samples were also performed throughout all of the experiments. The detection limits of Cd, Co, Cr, Cu, Fe, Mn, Ni and Pb were 0.01, 0.1, 1, 0.2 mg/kg, 0.01 (%), 1, 0.1 and 0.1 mg/kg, respectively.

### Single element pollution indices

Single element pollution indices, which give information about how an individual element is concentrated at a site of interest relative to a background were used to evaluate metal contamination. These include contamination factor (C*f*), enrichment factor (E*f*) and geo-accumulation index (Igeo).

C*f* is a ratio of an element in sample to the background site value or an established criterion for that metal (*Equation 1*).^30^,^31^

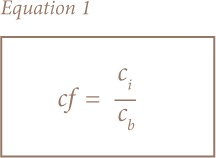
Where C_f_ is contamination factor, C_i_ is the concentration of metal of interest at a site and C_b_ is the concentration of the same metal at a control site. Four classes were established as follows (*Table 1*).^30^,^31^

**Table 1:** Thresholds for Soil and Sediment Quality Classification for Single Element Indices

Class	Qualification	*C*_*f*_	E*f*	Igeo
0	Unpolluted	C_*f*_ < 1	E*f*<2	Igeo < 1
1	Slightly			1 < Igeo <2
2	Moderately	1<C*f*<3	2 < E*f*< 5	2 < Igeo <3
3	Moderately-Heavily/Severely			3 < Igeo <4
4	Severely	3 <C*f*< 6	5 < E*f*< 20	4 < Igeo <5
5	High	C*f*>6	20 <E*f*< 40	
6	Extreme		E*f*> 40	Igeo > 5

Abbreviations: C*f* contamination factor; E*f* enrichment factor; Igeo, geo-accumulation index (Adapted from Brady et al., 2015)^30^

Enrichment factors of elements in the soil and sediment were determined by comparing the concentration of each element against the concentration of a control sample element to determine possible sources, i.e. crustal/geogenic/lithologic or anthropogenic (e.g., Fe, aluminium (Al) and Mn) in a given sample (*Equation 2*). An enrichment factor >1 is an indication of additional anthropogenic sources of the element of interest. For this study, Fe was used as the normalization element.

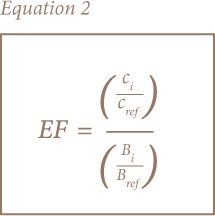
where (C_i_/C_ref_) is the ratio of concentration between element of interest and a reference element in the sample and (B_i_/B_ref_) is the ratio of concentration between element of interest and a reference element in the background sample. Generally, five contamination categories were associated with the enrichment factor.^31^

The Igeo proposed by Muller was used to describe metal contamination by comparing current concentrations with pre-industrial levels.^32^ This was calculated using Equation 3 and the comparison was based on seven classes of qualification.^31^

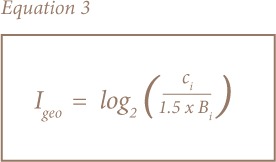
where Ci is the measured concentration of the examined metal in sediment, and Bi is the geochemical background concentration or reference value of the metal. A factor of 1.5 was used to account for the possible variations in background values for a given metal in the environment as well as very small anthropogenic influences. Seven grades of qualification were distinguished by Muller.^32^

### Multi-element pollution indices

Multi-element pollution indices were used for the assessment of soil and sediment due to the limitations of single element pollution indices.^23^,^30^,^33^ The most common and widely used are the contamination degree (C_d_) and pollution index (PI) developed by Hakanson and Nemerow, respectively, while the modified pollution index (MPI) was later proposed by Brady et al. and uses enrichment factors instead of contamination factors in its calculation.^23^,^30^,^34^ This takes into account the background concentrations and the complex, non-conservative behavior of sediments. Equations 4–6 show how the modified degree of contamination, PI and MPI were calculated.

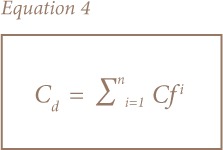


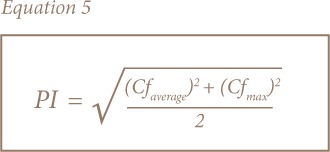


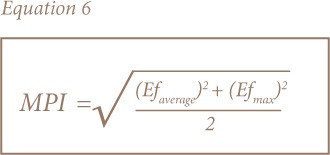
where Cf_i_, Cf_average_, Ef_average_, Cfmax and Ef_max_ represent the contamination factor for an individual element, average of contamination factors, average of enrichment factors, maximum contamination factor and maximum enrichment factor, respectively. The corresponding categories are presented in Table 2.

**Table 2:** Thresholds for Soil and Sediment Quality Classification for Multi-element Indices

Class	Qualification	C_*d*_	PI	MPI
0	Unpolluted	C_*d*_<1.5	PI <0.7	MPI<1
1	Slightly polluted	1.5< C_*d*_<2	0.7<PI<1	1<MPI<2
2	Moderately polluted	2≤ C_d_≤4	1<PI<2	2<MPI<3
3	Moderately-heavily polluted	4≤C_d_≤8	-	3<MPI<5
4	Severely polluted	8≤C_d_≤16	2<PI<3	5<MPI<10
5	Heavily polluted	16≤C_d_≤32	PI>3	MPI>10
6	Extreme polluted	C_*d*_>32	-	-

Abbreviations: C_*d*_, contamination degree; PI, pollution index; MPI, modified pollution index (Adapted from Brady et al., 2015)^30^

To quantitatively express the potential ecological risk of a given contaminant in a given area, the ecological risk factor (Er^i^) is expressed as:

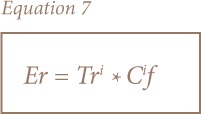


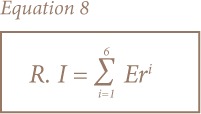



Where Tr^i^ is the toxic-response factor for a given substance; Hakanson defined Tr as a “toxic-response factor” for a given substance and demonstrated values for Cd, Cu, Pb, Ni, Cr, Zn, Mn of 30, 5, 5, 5, 2, 1, 1, respectively. C^i^*f* is the contamination factor, Er^i^ is the ecological risk factor for a given element, and RI is the requested potential ecological risk index for the location.

The following categories were used to describe the risk factor:

Er^i^ < 40 = low potential ecological risk;
40 ≤ Er^i^ ≤ 80 = moderate potential ecological risk;
80 ≤Er^i^ ≤ 160 = considerable potential ecological risk;
160 ≤ Er^i^ ≤ 320 = high potential ecological risk;
Er^i^ 320 > =very high ecological risk at hand for the substance in question.

## Results

**Figure 3:**
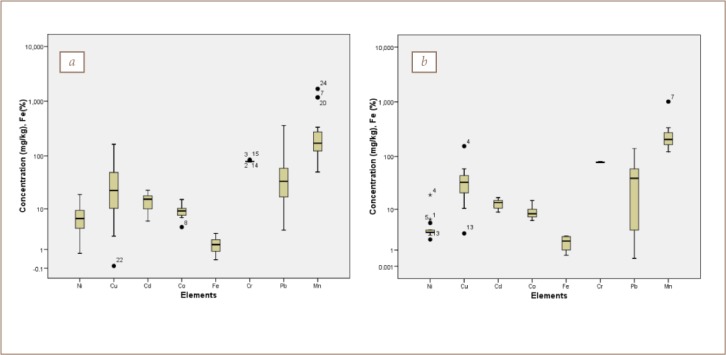
Statistical summary of elements in (a) soil and (b) sediment in the study area

The metals evaluated in the present study included Cd, Cr, Co, Cu, Ni, Pb, Mn and Fe. The geochemical results of soils and sediments in the study area are presented in Tables 3 and 4, respectively. The concentrations of all of the heavy metals in soils and sediments showed a relatively wide range of values, except for Cr. The mean concentrations of Cr, Cu, Mn, Ni, Pb, Co, Cd and Fe for soil and sediment samples were: 79.90, 40.70, 324.25, 6.87, 66.10, 9.06, 14.27 mg kg^−1^ and 1.50% and 78.62, 39.78, 266.75, 4.58, 40.62, 8.35, 12.94 mg kg^−1^ and 1.74%, respectively (*Figure 3*). A statistical summary of the metal contents including the mean value, standard deviation, range and background value is presented in Table 5. Approximately 25% of Cu, 63% of Cd and 45% of Pb, 12% of Mn, 8% of Co, and 8% of Fe concentrations were above control values, while those of Ni and Cr were generally lower than the control value in the study area. A comparison of the metal concentrations in both media with the control sample showed traces of the heavy metals in soils and sediments.

**Table 3:** Elemental Concentrations in Study Area Soil

Sample No	Ni (mg/kg)	Cu (mg/kg)	Cd (mg/kg)	Co (mg/kg)	Fe (%)	Cr (mg/kg)	Pb (mg/kg)	Mn (mg/kg)
S1	19.1	163.5	12.0	15.2	0.8	80.5	151.2	316.5
S2	11.0	38.0	6.8	9.0	1.9	84.8	363.9	245.0
S3	9.5	27.5	6.7	9.1	1.5	84.5	235.8	248.5
S4	8.1	10.0	13.6	12.1	0.3	79.7	11.6	284.0
S5	5.2	10.5	16.3	9.2	0.6	79.8	7.5	336.5
S6	4.0	5.0	19.7	10.4	0.7	79.1	4.4	260.5
S7	8.3	10.0	20.0	10.0	1.0	78.2	20.3	1175.0
S8	0.7	33.0	20.8	4.1	2.0	78.7	32.0	50.5
S9	6.9	8.5	22.9	7.9	2.0	78.0	10.4	88.0
S10	9.1	30.0	16.0	10.4	1.2	78.7	30.1	124.5
S11	9.9	28.5	19.3	12.4	1.9	78.9	23.6	176.0
S12	3.7	120.5	17.4	8.7	2.0	77.6	118.9	165.0
S13	3.3	45.5	15.1	7.5	0.9	77.8	43.0	271.5
S14	5.5	12.0	5.6	6.8	0.8	84.9	35.9	190.0
S15	6.4	12.0	9.0	8.4	1.7	84.6	18.5	74.5
S16	6.2	18.5	8.8	7.3	1.4	81.2	51.1	122.0
S17	10.7	115.0	10.5	9.1	1.4	79.9	57.1	153.0
S18	4.3	11.0	9.5	6.8	1.3	80.3	21.3	142.5
S19	2.1	2.5	10.5	6.6	0.6	80.4	3.5	164.5
S20	3.3	68.0	16.5	12.5	2.0	78.2	61.5	1180.0
S21	8.2	18.0	16.6	8.8	2.0	78.7	15.8	107.0
S22	10.0	0.0	15.8	9.5	2.9	78.0	50.3	124.0
S23	6.1	54.0	12.8	9.6	2.3	77.9	54.1	98.0
S24	3.7	127.5	18.7	6.9	2.7	77.7	107.7	1685.0
Control Sample	28.0	40.0	10.5	18.0	1.7	85.0	40.0	780.0

**Table 4:** Elemental Concentrations in Study Area Sediments

Sample No	Ni (mg/kg)	Cu (mg/kg)	Cd (mg/kg)	Co (mg/kg)	Fe (%)	Cr (mg/kg)	Pb (mg/kg)	Mn (mg/kg)
SS1	6.2	33.0	8.8	10.0	1.4	81.2	51.1	122.0
SS2	3.1	20.0	9.2	5.9	2.6	77.8	3.6	129.5
SS3	3.2	33.5	15.0	6.4	2.6	78.0	59.3	160.0
SS4	19.1	155.0	16.9	15.0	0.8	80.5	141.0	316.5
SS5	5.2	10.5	16.3	11.0	0.6	79.8	68.9	336.5
SS6	3.2	43.5	15.3	9.9	1.7	78.4	47.4	242.0
SS7	2.7	59.0	14.7	9.9	2.1	78.3	79.3	1010.0
SS8	2.8	49.0	14.5	10.5	2.1	78.2	52.7	274.0
SS9	3.6	25.5	13.3	7.2	2.2	77.4	32.6	203.5
SS10	3.2	44.5	13.1	8.3	1.7	77.9	9.2	211.5
SS11	3.2	25.0	10.6	7.0	2.5	77.4	0.4	187.5
SS12	3.2	21.0	13.7	7.8	2.6	77.7	7.5	169.0
SS13	2.1	3.0	10.5	8.0	0.6	80.4	3.5	164.5
SS14	3.3	41.0	11.6	6.7	1.0	77.9	2.2	208.0
Control Sample	28.0	40.0	10.5	18.0	1.7	85.0	40.0	780.0

**Table 5:** Summary of the Elemental Results for Soil and Sediments in the Study Area

Element	Mean ± Standard Deviation (mg/Kg)		Range (mg/Kg)	

	Soil	Sediments	Soil	Sediment
Ni	6.9±3.9	4.6± 4.3	0.7–19.1	2.1–19.1
Cu	40.4± 46.5	39.8± 38.9	0.0–163.5	2.7–155.0
Cd	14.3± 5.0	12.9± 2.6	5.6–22.9	8.8–16.9
Co	9.1± 2.4	8.4±2.4	4.1–15.2	5.9–15.0
Fe	1.5± 0.7	1.7 ±0.7	0.3–2.9	0.6–2.6
Cr	79.9± 2.4	78.6±1.3	77.6–84.9	77.4–81.2
Pb	66.1± 87.3	40.6±42.2	3.5–363.9	0.4–141.0
Mn	324.3± 411.5	266.8 ±223.3	50.5–1685.0	122.0–1010.0

The spatial variation of heavy metals in stream sediments was lower in the samples from points before the river entered the industrial area (SS1-2 and SS6-7) (*Figure 1 and Supplemental Material*) and increased on entering the industrial area, reaching maximum values at SS4 (where most of the industrial effluents are discharged directly from a common drainage into the river body). This implies that there was enrichment of the heavy metals in the sediments by the industrial effluents in the area. This was corroborated by the results of the E*f*, where Cu, Cd, Co and Pb were observed to be significantly enriched at points within the industrial region.

A comparison of the metal concentrations with other cities in Nigeria like Lagos and Benin where similar studies were conducted revealed higher concentrations for most of the heavy metals than what was measured, especially for Cd, Cu and to some extent Pb (*Table 6*). On a global scale, the measured metals concentrations were also comparable to those measured in Hunan, China, and Brisbane, Australia (*Table 6*).

**Table 6:** Heavy Metals Concentrations Across Different Locations

**Elements**	Ibadan, Nigeria	Lagos, Nigeria	Benin, Nigeria	Brisbane, Australia	Hunan Province, China
**Ni**	0.65–19.10	-	-	20.0–34.00	57–304
**Cu**	0.00–163.5	28.0–153.0	5–30	20–110.0	42–99
**Cd**	6.0–23.0	0.20–2.10	0–3.4	0.6–0.9	1.85–25.20
**Co**	4.10–15.15	-	-	17–29	21–103
**Fe**	0.31–2.86	-	0.5–3.0	5.1–7.7	34.06–92.62
**Cr**	77.55–84.85	-	-	82–332	65–160
**Pb**	3.50–363.85	20.00–148.00	1–14	25–126	46–1735
**Mn**	50.50–1685.00	189.00–2024.00	10–54	319–1143	-
Reference	Present study	Olatunji et al., 2009^5^	Enuneku et al., 2017^24^	Duodu et al., 2016^33^	Zhang et al., 2011^6^

### Soil and sediment characteristics

**Figure 4:**
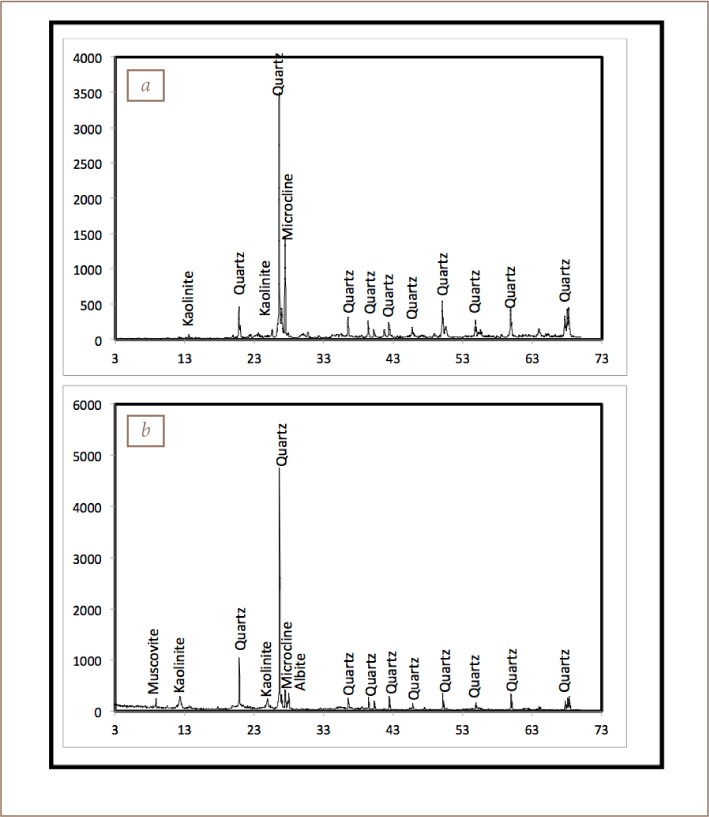
Diffractograms of selected (a) soil and (b) sediment samples in the study area

The mineralogical results for the soil and sediments are presented in Figure 4 and Table 7, respectively. The dominant minerals in both media were quartz (78–89%), microcline (5–22%), albite (4–7%), mica (0–5%), with traces of kaolinite in soil. A similar mineralogical composition was also observed in sediment with quartz (62–78%), albite (3–15%), microcline (2–20%), and mica (0–7%) except for kaolinite, which was higher, reaching 20% in some cases. These mineral assemblages were similar to those in a previous study in the area.^4^

**Table 7:** X-Ray Diffractometry Semi-Quantitative Mineralogical Abundance

Minerals	Soils (%)	Sediments (%)
Quartz	78–89	62–78
Biotite	0–3	0–2
Muscovite	0–5	0–7
Microcline	5–22	2–20
Albite	4–7	3–15
Amphibole	<1	0–1
Kaolinite	0–5	5–20
Unidentified Minerals	-	6

The mineralogy of the soil and sediment samples confirmed that the samples were mostly derived from weathering of the underlying geology, reflecting the end product of weathering.^4^,^12^,^28^

### Heavy metal sourcing

**Figure 5:**
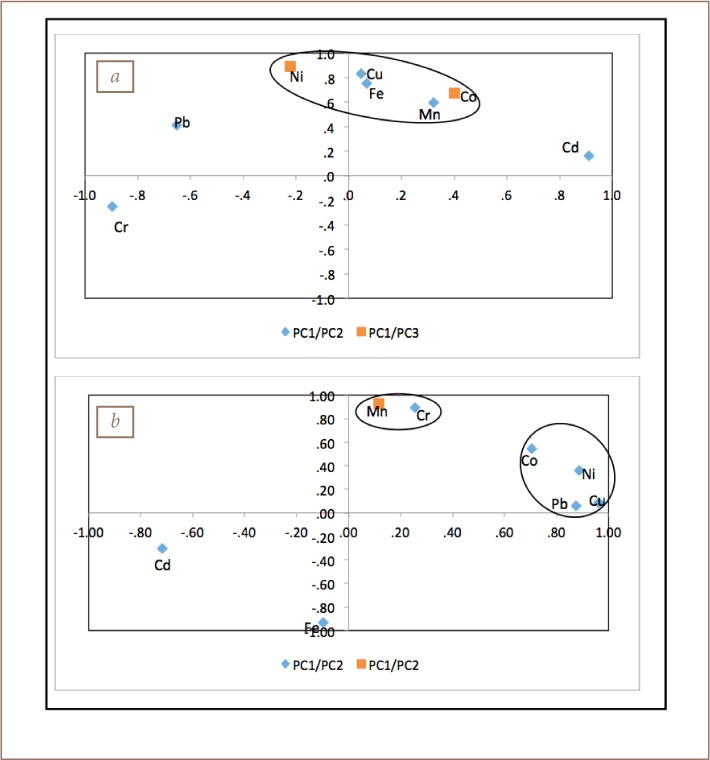
Principal component analysis loading plot of (a) soil (PC 1 vs PC 2/ PC 3) and (b) sediment (PC 1 vs PC 2/ PC 3)

The results were subjected to principal component (PC) analysis in order to constrain the sources of heavy metals in both the soil and sediment by applying varimax rotation with Kaiser normalization (*Tables 8 and 9, Figure 5*). The results indicated that there were three components with Eigen values higher than 1.0 responsible for 74.3% (soil) and 87.8% (sediment) of the total variance in the analysis.

**Table 8:** Principal Component Analysis of Elements in Soil

Elements	PC 1	PC 2	PC 3	Communalities
Ni	−0.2	0.0	0.9	0.8
Cu	0.1	0.8	0.3	0.7
Cd	0.9	0.2	0.3	0.9
Co	0.4		0.7	0.7
Fe	0.1	0.8	0.2	0.6
Cr	−0.9	−0.3	0.1	0.9
Pb	−0.7	0.4	0.1	0.8
Mn	0.3	0.6	0.2	0.5
Eigen value	2.5	2.0	1.5	
% variance	30.8	24.9	18.7	
Cumulative %	30.8	55.6	74.3	

Abbreviations: PC, principal component

**Table 9:** Principal Component Analysis of Elements in Sediment

Elements	PC 1	PC 2	PC 3	Communalities
Ni	0.9	0.4	−0.1	0.9
Cu	1.0	0.1	0.2	1.0
Cd	−0.7	−0.3	0.4	0.8
Co	0.7	0.5	0.3	0.9
Fe	−0.1	−0.9	0.0	0.9
Cr	0.3	0.9	−0.1	0.9
Pb	0.9	0.1	0.2	0.8
Mn	0.1	0.0	0.9	0.9

Eigen value	3.6	2.2	1.2	
% variance	44.7	27.5	15.6	
Cumulative %	44.7	72.2	87.8	

Abbreviations: PC, principal component

For soil, PC 1 had the strongest factor with positive loading of Cd (0.9) accounting for 30.8% of the total variance (*Table 5*). PC 2 had Cu, Fe and Mn, accounting for 24.9% of the total variance. PC 3 had Ni and Co, accounting for 18.7% of the total variance. For sediments, PC 1 had positive loadings of Ni, Cu, and Pb, accounting for 44.7% of the total variance. PC 2, which accounted for 27.5% of the total data variance, had Co, Cr, and Fe, while PC 3 had Mn with a very high factor loading of 0.93, accounting for 15.6% of the total variance.

The metal association can be attributed to two broad sources: anthropogenic (industrial and related activities) and geogenic (locked in the mineralogy of the soil and sediments). Anthropogenic sources were defined by the groupings in PC1 for both soils and sediments. The industrial activities use a variety of chemicals as raw materials and some of their products generate heavy metals that are disposed indiscriminately within the immediate environment and surrounding river channels.^18^,^19^,^20^ The geogenic contribution to metals concentrations is defined by PC 2 and 3 of both soil (cumulative % of 44) and sediments (cumulative % of 43.1).

## Discussion

### Single-element indices

**Figure 6:**
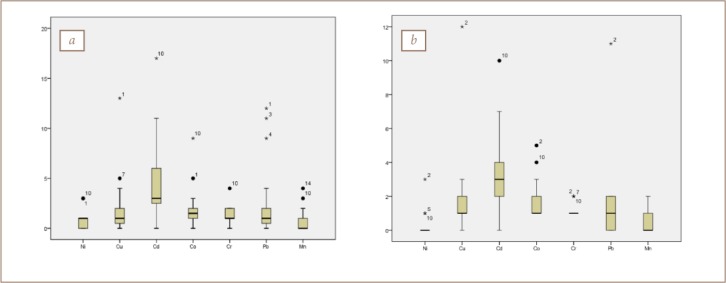
Summary of enrichment factor of elements in (a) soil and (b) sediment

The enrichment factor of most of the metals showed depletion to significant enrichment at various sites (*Table 10, Figure 6*). For soil samples, Cu, Cd, Co and Pb showed significant enrichment at (S1 and S17), (S19, S4–S7, S9–S10 and S13), (S1 and S4), (S1–S3) and (S4), respectively; while for sediment, only Cu (SS13) and Cd (SS1–SS4, SS5 and SS13) showed moderate to significant enrichment, respectively.

**Table 10:** Geoaccumulation Index for Soil and Sediment

The calculated Igeo (*Table 11*) indicated that all of the elements were “unpolluted to slightly polluted” in both the soils and stream sediments, except for Cd. However, Pb in samples S10 and S11 was classified as severely polluted.

**Figure 7:**
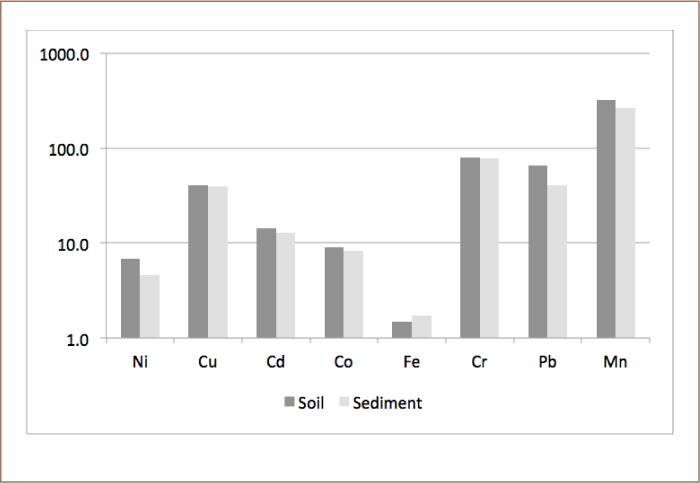
Contamination factor of soil and sediment

The calculated contamination factor of heavy metals also revealed that most of the metals were within low to considerable contamination classes with (S1, S17 and S12), (S6, S7–S9, S11, S13, S20, S21 and S24), and (S1–S3 and S13) being highly contaminated with Cu, Cd and Pb, respectively (*Figure 7*).

These single pollution indices shed light on the quality of soil and sediment in the study area, and indicate that most of the elements were unpolluted to moderately polluted with regard to Igeo and EF. Most of the moderately polluted points fell within the industrial region. However, some of the sites in the industrial region showed significant enrichment, especially for Cd, Pb and Cu. The contamination factor showed significant to heavily contaminated status for most of the sampling points in the industrial area, but S24 was also heavily contaminated for Pb and Cd because the site was closer to a major road intersection with a large traffic volume.

### Multi-element indices

**Figure 8:**
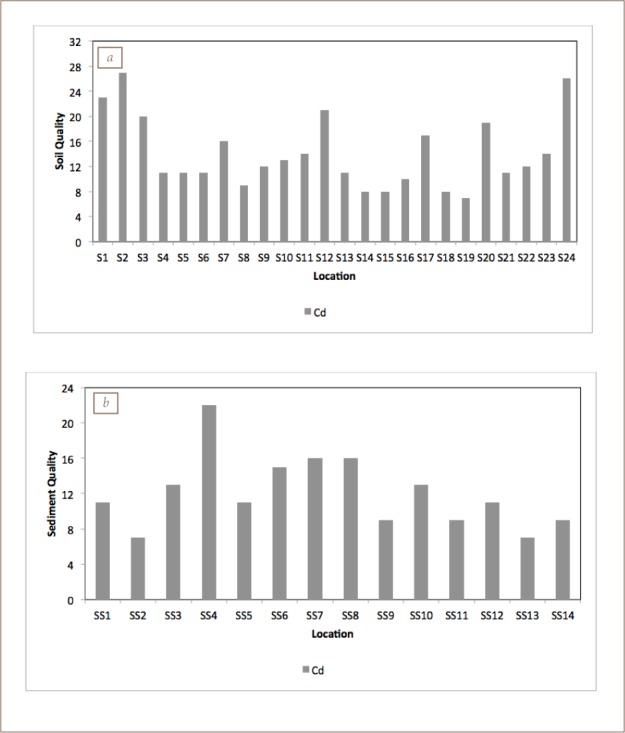
Contamination degree of (a) soils and (b) sediments

**Figure 9:**
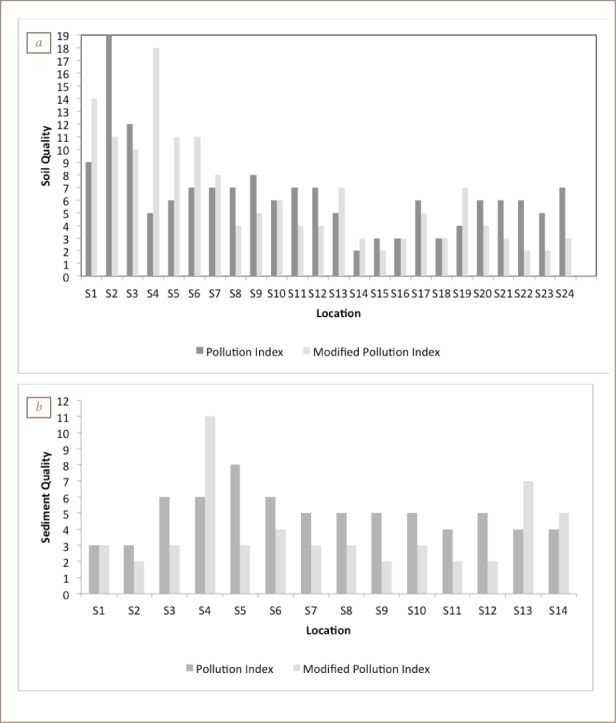
Pollution index and modified pollution index chart for (a) soil and (b) sediment samples

The degree of contamination results (*Figure 8*) indicated a “low to moderate degree” of contamination, with about 70% of sites having an index lower than 16. Samples S1, S2, S3, S12, S17, S20 and S24 showed a considerable degree of contamination. On the basis of both the PI and MPI (*Figure 9*), the soils and sediments can be described as “severely polluted”.

It was observed that while contamination degree underestimated the risk posed by both the soil and sediment to the effect that none of the sites were found to be heavily polluted, the PI overestimated the risk at all sites except at S9, S12, S70 and S71, which were deemed to be “moderately polluted” due to the high and lower trigger values, respectively. Thus, PI seemed to have an advantage over the other indices, as low trigger values effectively classify sediments that pose high risk and warrant further examination to identify the sources of contamination.^31^ The MPI clearly distinguish between “slightly polluted” (S15, S22 and S23), “moderately polluted” (S14, S16, S18, S21 and S24), “moderately-heavily polluted” (S8, S9, S18, S11, S12, S17 and S20) and “severely polluted” (S7, S10, S13, and S19) samples. However, only 21% of the sites (S1, S2, S4, S5 and S6) were heavily polluted.

### Ecological risk assessment

**Figure 10:**
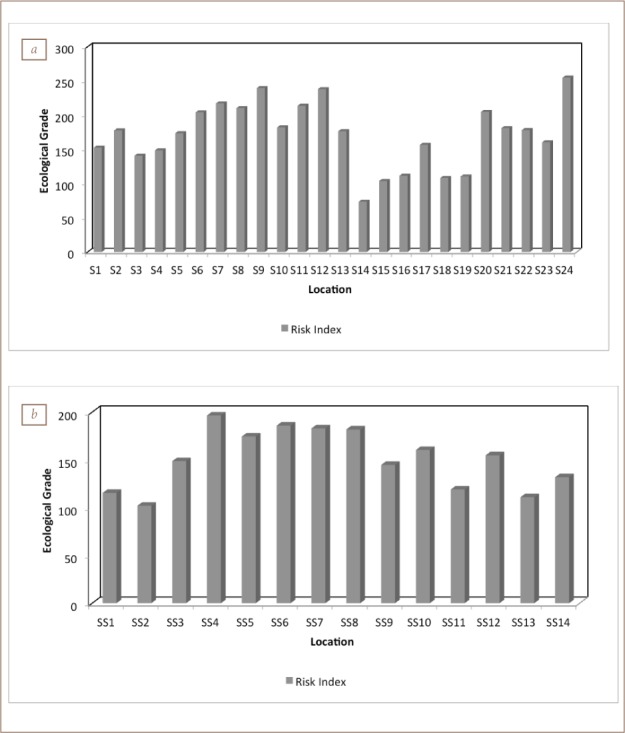
Ecological risk index of heavy metals in (a) soil and (b) sediment

The potential Er^i^ for the heavy metals in soil and sediments in the study area showed an order of Cd > Pb > Cu > Ni > Cr > Mn (*figure 10*). Generally, most of the locations had low potential Er^i^ values for all the heavy metals in the soil and sediment.

All of the sampling points in the industrial region demonstrated a strong potential Er^i^ for Cd, Pb and Cu. Cadmium was the key influencing factor causing the potential Er^i^.

## Conclusion

The present study examined the concentrations and contamination of heavy metals in soils and sediments of Ibadan industrial area. The results demonstrated that there had been considerable enrichment in the levels of Cd, Pb and Cu in soil and sediment in the study area. The source of these enrichments was mainly attributed to industrial and associated geogenic activities. The sampling points within the industrial area demonstrated a strong potential ecological risk for Cd, Pb and Cu. Continuous monitoring of the industrial area for heavy metal contamination and policies that support reduction of contamination are needed. Further study is also recommended due to the uncertainty inherent in comparing site samples to a single control sample in the present study.
