# Supplementary material for: Heavy Metal Contamination and Ecological Risk Assessment in Soils and Sediments of an Industrial Area in Southwestern Nigeria
Source: J Health Pollut. 2018 Aug 21;8(19):180906. doi: 10.5696/2156-9614-8.19.180906 (PMC6257164; doi:10.5696/2156-9614-8.19.180906)

Supplemental Material

Variation diagram of selected elements in stream sediment from upstream to downstream.

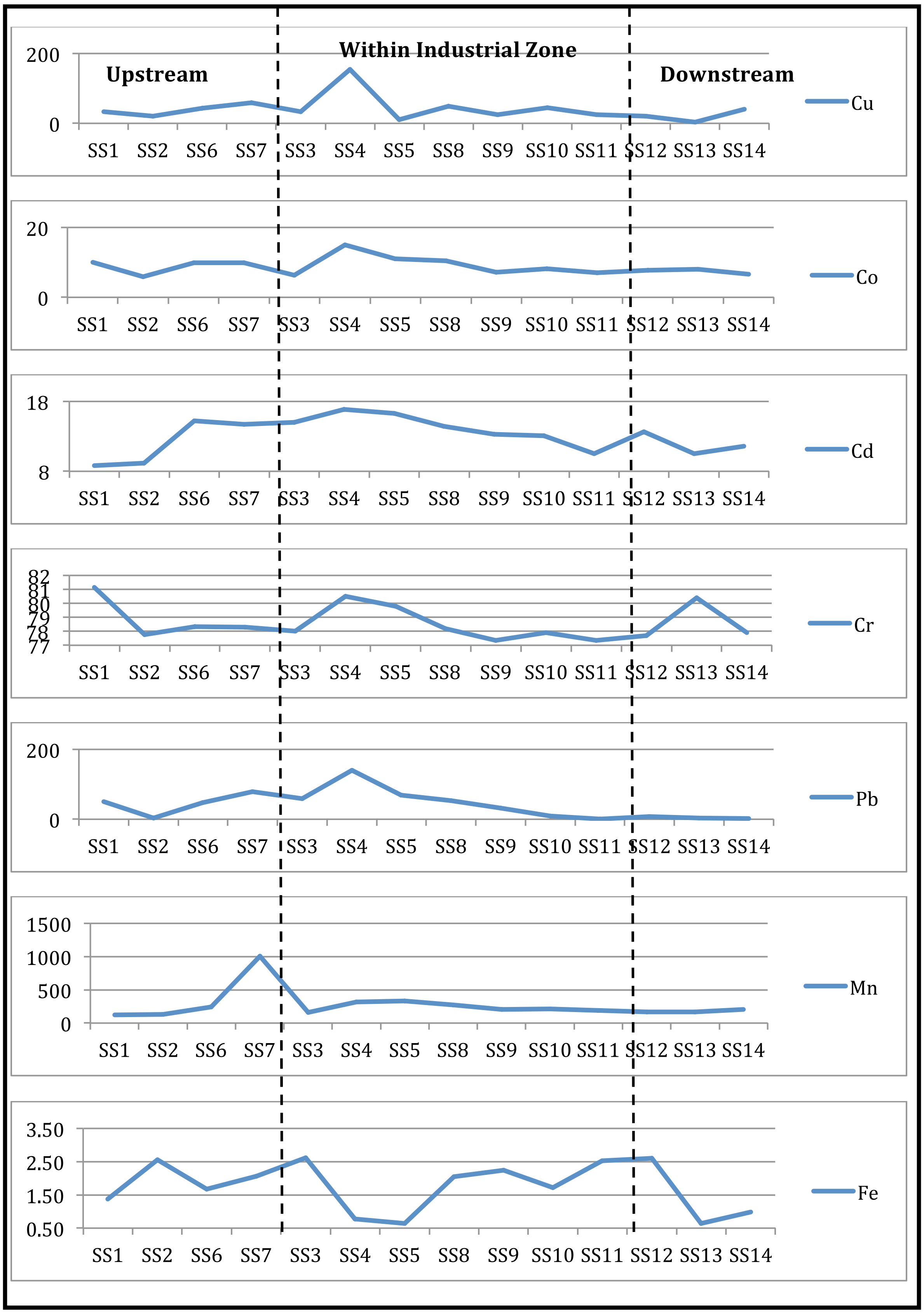

Supplement: Supplementary file 1 [file Kolawole_Supplemental_Material.pdf]
